# Supplementary material for: Cortical signatures of sleep are altered following effective deep brain stimulation for depression
Source: Transl Psychiatry. 2024 Feb 20;14:103. doi: 10.1038/s41398-024-02816-z (PMC10879134; doi:10.1038/s41398-024-02816-z)
Supplement: Supplementary file 1 — Supplemental Material [file 41398_2024_2816_MOESM1_ESM.pdf]

Supplementary Figures

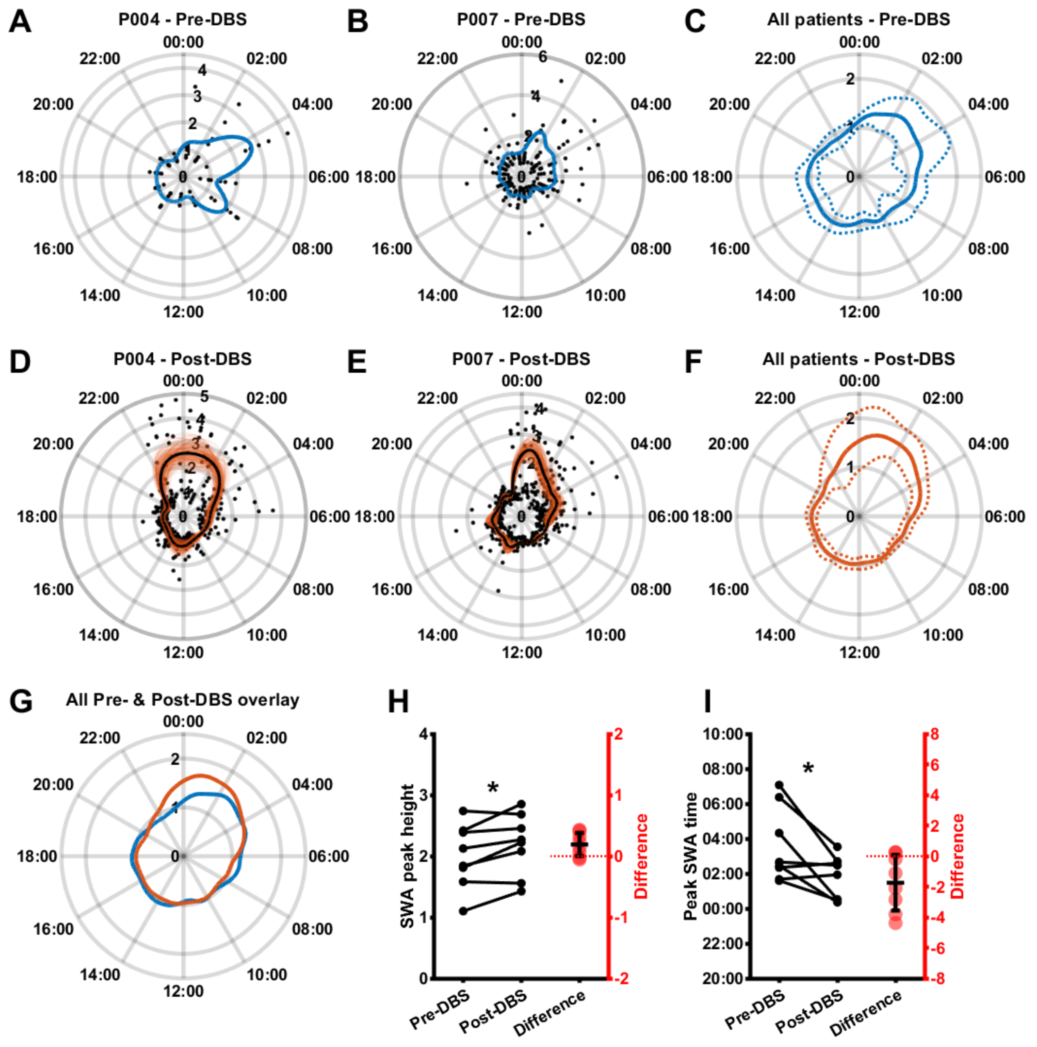

Figure S1: Random resampling of the Post-DBS phase confirms that slow-wave activity is more temporally consistent and occurs earlier in the night after SCC DBS

A, B: Log-transformed and median-normalized SWA power (mean across both hemispheres; black) and a smoothing spline fit (blue) around the 24 hours of the day in the Pre-DBS phase for two example patients. **C:** Mean (solid line) plus and minus standard deviation (dotted lines) across patients of Pre-DBS time-of-day fits as illustrated in A and B. **D, E:** Log-transformed and median-normalized SWA power (mean across both hemispheres; black) and 1000 overlaid smoothing spline fits (orange) as well as their mean (black) around the 24h of the day in the Post-DBS phase for the same example patients shown in A and B. **F:** Mean (solid line) plus and minus standard deviation (dotted lines) across patients of the mean resampled Post-DBS time-of-day fits as illustrated in D and E. **G:** Mean Pre-DBS (blue line) and resampled Post-DBS (orange line) time-of-day fits across patients highlighting increased SWA in the late evening and early night in the Post-DBS phase. **H:** Pre-DBS vs. resampled Post-DBS median height of the greatest night-time SWA peak as estimated by the time-of-day fit (normalised to median; Pre-DBS: 2.00±0.53, Post-DBS: 2.41±0.40, $t(7) = -3.81$, $p = 0.0066$, $n = 8$). **I:** Pre-DBS vs. resampled Post-DBS median time of day of the greatest night-time SWA peak as estimated by the time-of-day fit (in 24h clock time; Pre-DBS: 03:34±02:08, Post-DBS: 01:19±01:33, $t(7) = 3.26$, $p = 0.0139$, $n = 8$).

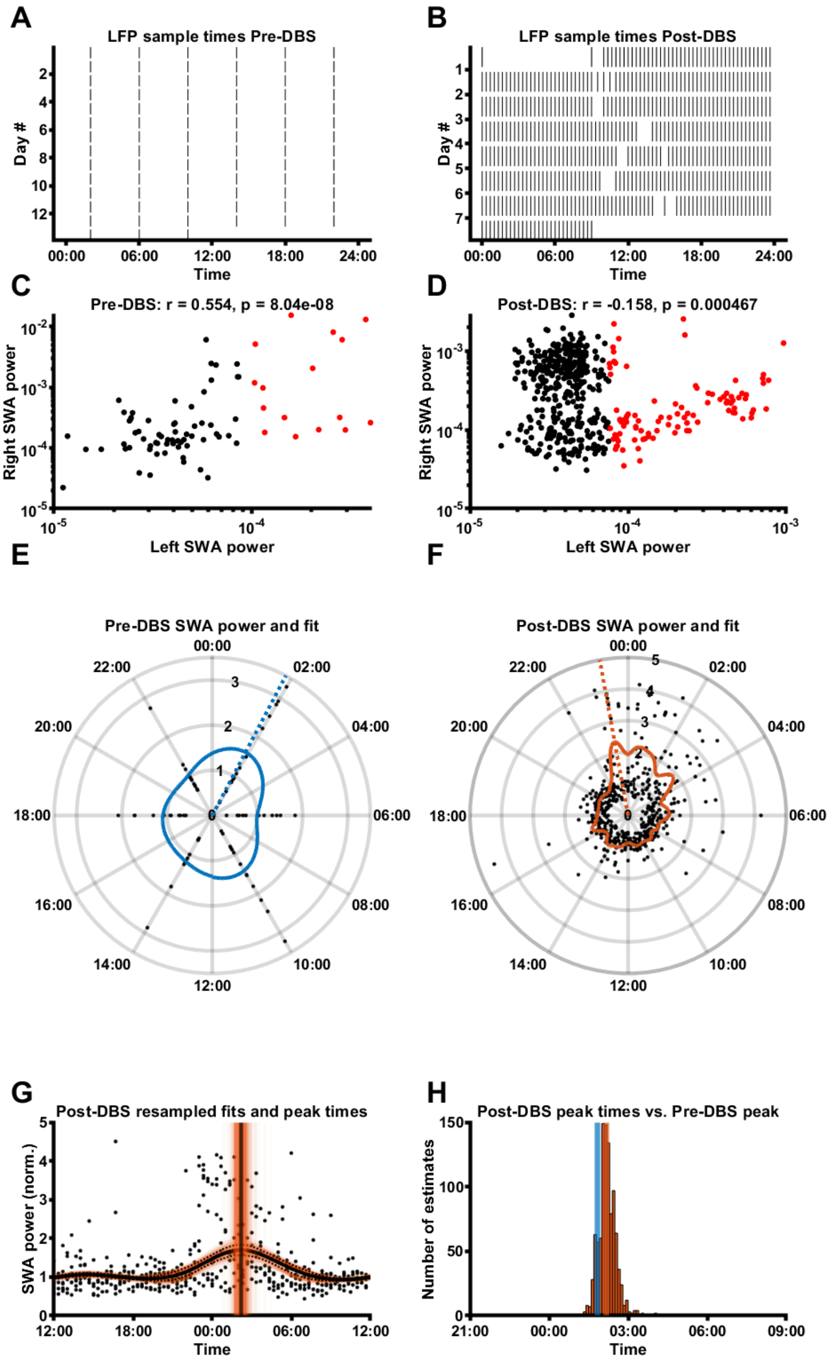

Figure S2: P001 SWA data overview

A, B: SCC LFP sample times in the Pre-DBS (A) and Post-DBS (B) phase. **C, D:** Correlation of left and right SCC SWA power Pre-DBS (C) and Post-DBS (D). **E, F:** SWA power (normalised to median) of all LFPs for this participant (mean across valid hemispheres), plotted around the 24h diurnal cycle. The coloured line represents a smoothing spline fit to the data; the dashed line represents the time of the maximum night-time (18:00-10:00) peak of the SWA fit. **G:** SWA power (normalised to median) of all LFPs for this participant (mean across valid hemispheres), with a mean±SD fit line (solid black line and dashed black lines) superimposed on 1000 fit lines (thin, orange) obtained through random re-sampling of Post-DBS data according to the sample times Pre-DBS (see Methods for details). Also indicated is the median fit peak estimate (black vertical line) superimposed on 1000 fit peak estimate lines (thin orange vertical lines). **H:** Distribution of fit peak time estimates obtained through the 1000 random resamples of the Post-DBS phase, with the median peak time estimate indicated with the orange vertical line and the fit peak time from Phase B indicated with the blue vertical line.

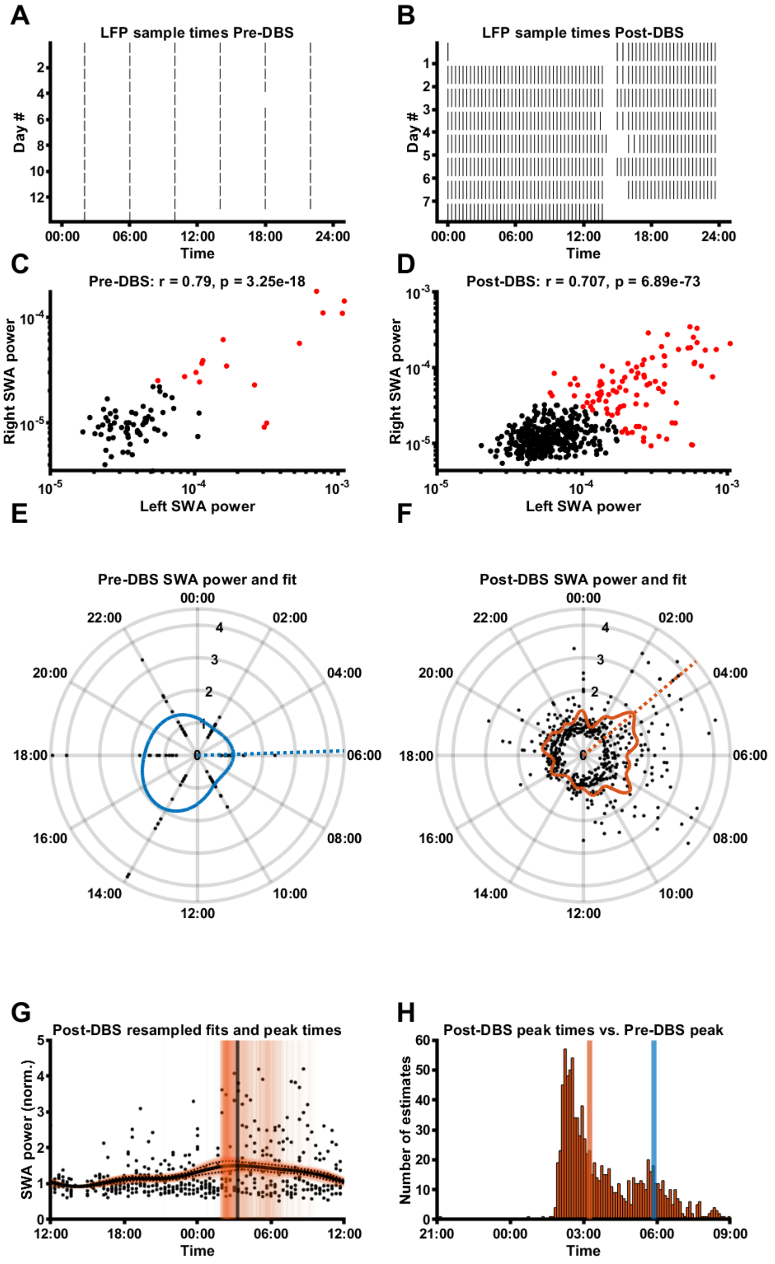

Figure S3: P002 SWA data overview

A, B: SCC LFP sample times in the Pre-DBS (A) and Post-DBS (B) phase. **C, D:** Correlation of left and right SCC SWA power Pre-DBS (C) and Post-DBS (D). **E, F:** SWA power (normalised to median) of all LFPs for this participant (mean across valid hemispheres), plotted around the 24h diurnal cycle. The coloured line represents a smoothing spline fit to the data; the dashed line represents the time of the maximum night-time (18:00-10:00) peak of the SWA fit. **G:** SWA power (normalised to median) of all LFPs for this participant (mean across valid hemisperes), with a mean±SD fit line (solid black line and dashed black lines) superimposed on 1000 fit lines (thin, orange) obtained through random re-sampling of Post-DBS data according to the sample times Pre-DBS (see Methods for details). Also indicated is the median fit peak estimate (black vertical line) superimposed on 1000 fit peak estimate lines (thin orange vertical lines). **H:** Distribution of fit peak time estimates obtained through the 1000 random resamples of the Post-DBS phase, with the median peak time estimate indicated with the orange vertical line and the fit peak time from Phase B indicated with the blue vertical line.

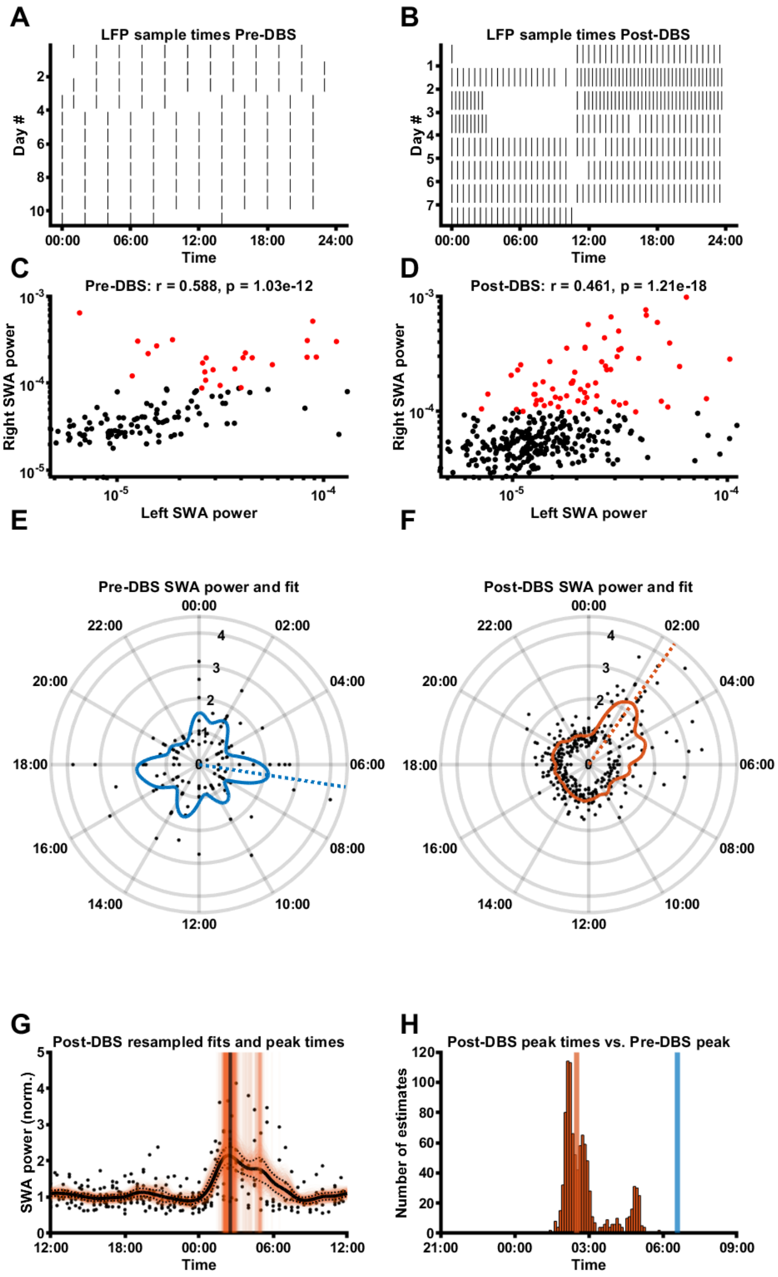

Figure S4: P003 SWA data overview

A, B: SCC LFP sample times in the Pre-DBS (A) and Post-DBS (B) phase. **C, D:** Correlation of left and right SCC SWA power Pre-DBS (C) and Post-DBS (D). **E, F:** SWA power (normalised to median) of all LFPs for this participant (mean across valid hemispheres), plotted around the 24h diurnal cycle. The coloured line represents a smoothing spline fit to the data; the dashed line represents the time of the maximum night-time (18:00-10:00) peak of the SWA fit. **G:** SWA power (normalised to median) of all LFPs for this participant (mean across valid hemispheres), with a mean±SD fit line (solid black line and dashed black lines) superimposed on 1000 fit lines (thin, orange) obtained through random re-sampling of Post-DBS data according to the sample times Pre-DBS (see Methods for details). Also indicated is the median fit peak estimate (black vertical line) superimposed on 1000 fit peak estimate lines (thin orange vertical lines). **H:** Distribution of fit peak time estimates obtained through the 1000 random resamples of the Post-DBS phase, with the median peak time estimate indicated with the orange vertical line and the fit peak time from Phase B indicated with the blue vertical line.

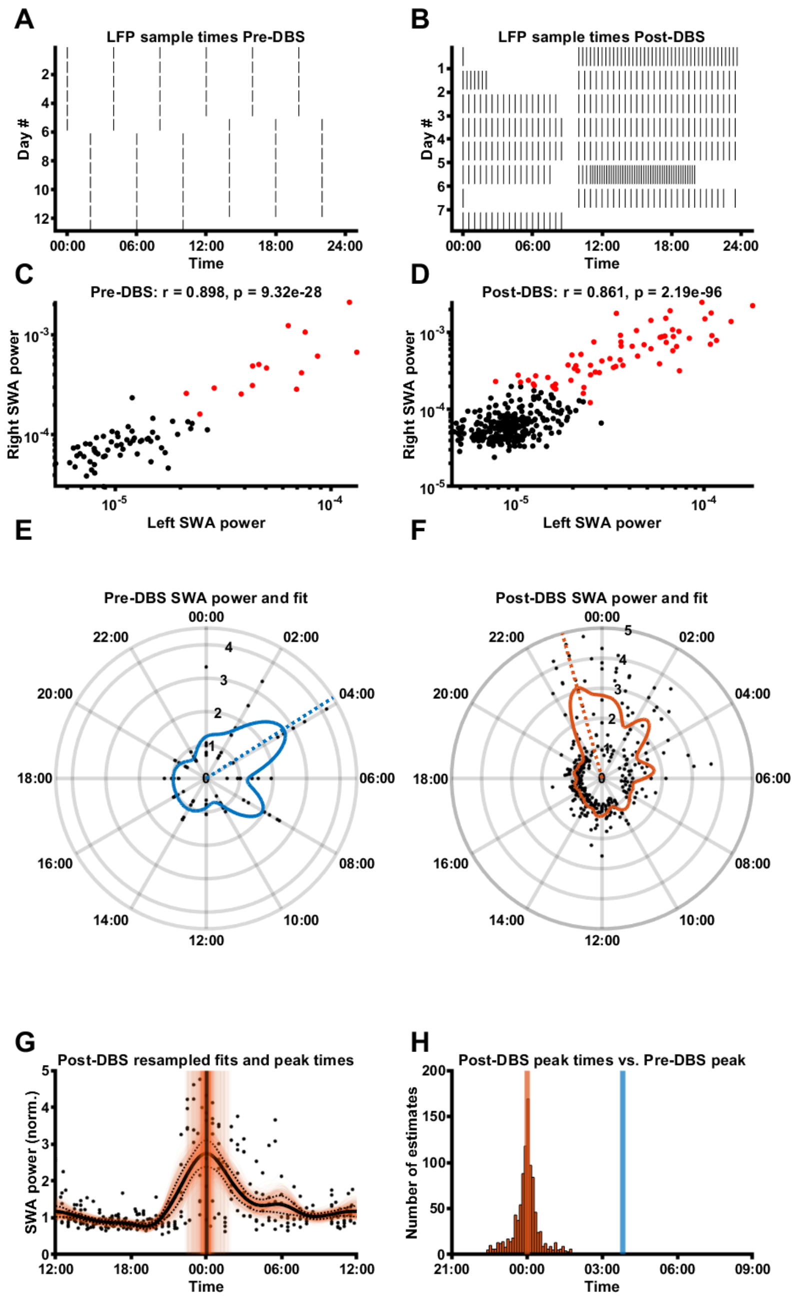

Figure S5: P004 SWA data overview

A, B: SCC LFP sample times in the Pre-DBS (A) and Post-DBS (B) phase. **C, D:** Correlation of left and right SCC SWA power Pre-DBS (C) and Post-DBS (D). **E, F:** SWA power (normalised to median) of all LFPs for this participant (mean across valid hemispheres), plotted around the 24h diurnal cycle. The coloured line represents a smoothing spline fit to the data; the dashed line represents the time of the maximum night-time (18:00-10:00) peak of the SWA fit. **G:** SWA power (normalised to median) of all LFPs for this participant (mean across valid hemispheres), with a mean±SD fit line (solid black line and dashed black lines) superimposed on 1000 fit lines (thin, orange) obtained through random re-sampling of Post-DBS data according to the sample times Pre-DBS (see Methods for details). Also indicated is the median fit peak estimate (black vertical line) superimposed on 1000 fit peak estimate lines (thin orange vertical lines). **H:** Distribution of fit peak time estimates obtained through the 1000 random resamples of the Post-DBS phase, with the median peak time estimate indicated with the orange vertical line and the fit peak time from Phase B indicated with the blue vertical line.

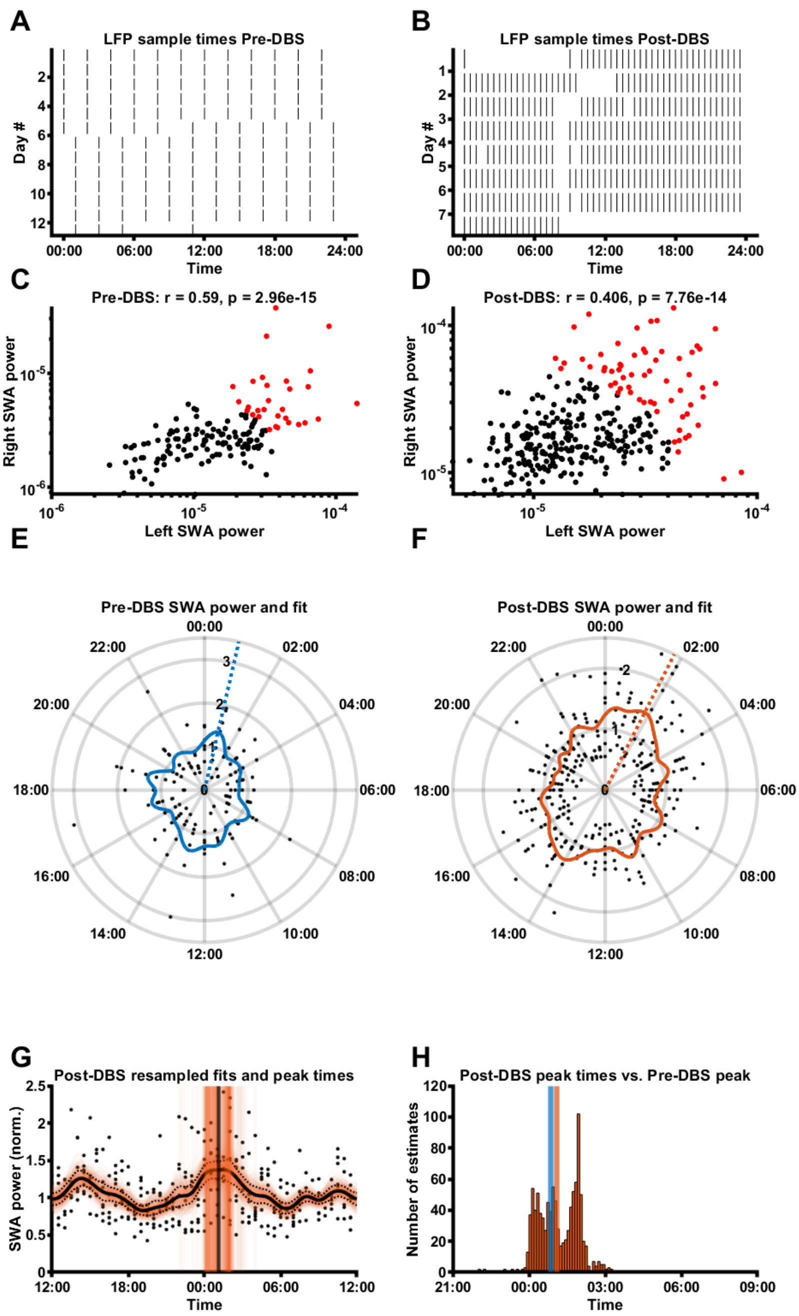

Figure S6: P005 SWA data overview

A, B: SCC LFP sample times in the Pre-DBS (A) and Post-DBS (B) phase. **C, D:** Correlation of left and right SCC SWA power Pre-DBS (C) and Post-DBS (D). **E, F:** SWA power (normalised to median) of all LFPs for this participant (mean across valid hemispheres), plotted around the 24h diurnal cycle. The coloured line represents a smoothing spline fit to the data; the dashed line represents the time of the maximum night-time (18:00-10:00) peak of the SWA fit. **G:** SWA power (normalised to median) of all LFPs for this participant (mean across valid hemispheres), with a mean±SD fit line (solid black line and dashed black lines) superimposed on 1000 fit lines (thin, orange) obtained through random re-sampling of Post-DBS data according to the sample times Pre-DBS (see Methods for details). Also indicated is the median fit peak estimate (black vertical line) superimposed on 1000 fit peak estimate lines (thin orange vertical lines). **H:** Distribution of fit peak time estimates obtained through the 1000 random resamples of the Post-DBS phase, with the median peak time estimate indicated with the orange vertical line and the fit peak time from Phase B indicated with the blue vertical line.

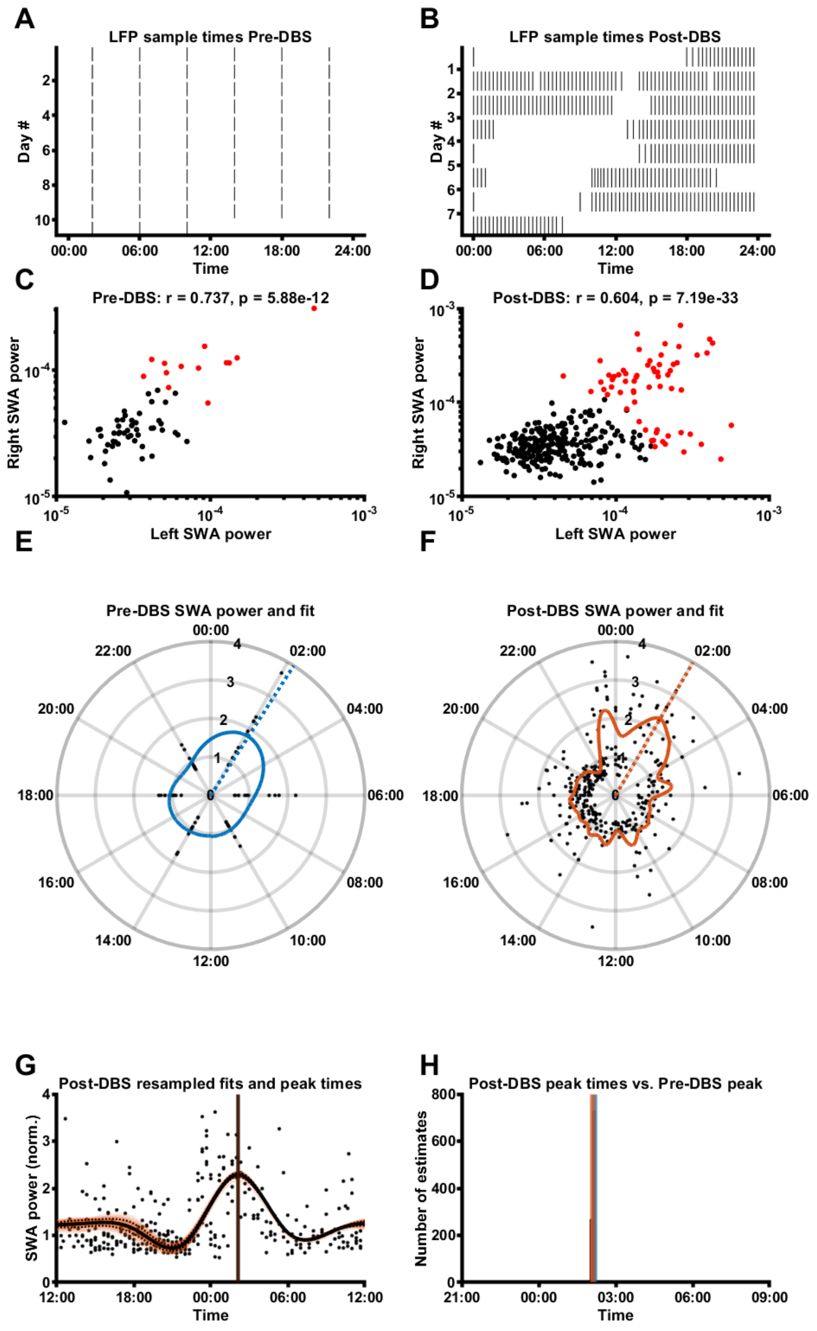

Figure S7: P006 SWA data overview

A, B: SCC LFP sample times in the Pre-DBS (A) and Post-DBS (B) phase. **C, D:** Correlation of left and right SCC SWA power Pre-DBS (C) and Post-DBS (D). **E, F:** SWA power (normalised to median) of all LFPs for this participant (mean across valid hemispheres), plotted around the 24h diurnal cycle. The coloured line represents a smoothing spline fit to the data; the dashed line represents the time of the maximum night-time (18:00-10:00) peak of the SWA fit. **G:** SWA power (normalised to median) of all LFPs for this participant (mean across valid hemispheres), with a mean±SD fit line (solid black line and dashed black lines) superimposed on 1000 fit lines (thin, orange) obtained through random re-sampling of Post-DBS data according to the sample times Pre-DBS (see Methods for details). Also indicated is the median fit peak estimate (black vertical line) superimposed on 1000 fit peak estimate lines (thin orange vertical lines). **H:** Distribution of fit peak time estimates obtained through the 1000 random resamples of the Post-DBS phase, with the median peak time estimate indicated with the orange vertical line and the fit peak time from Phase B indicated with the blue vertical line.

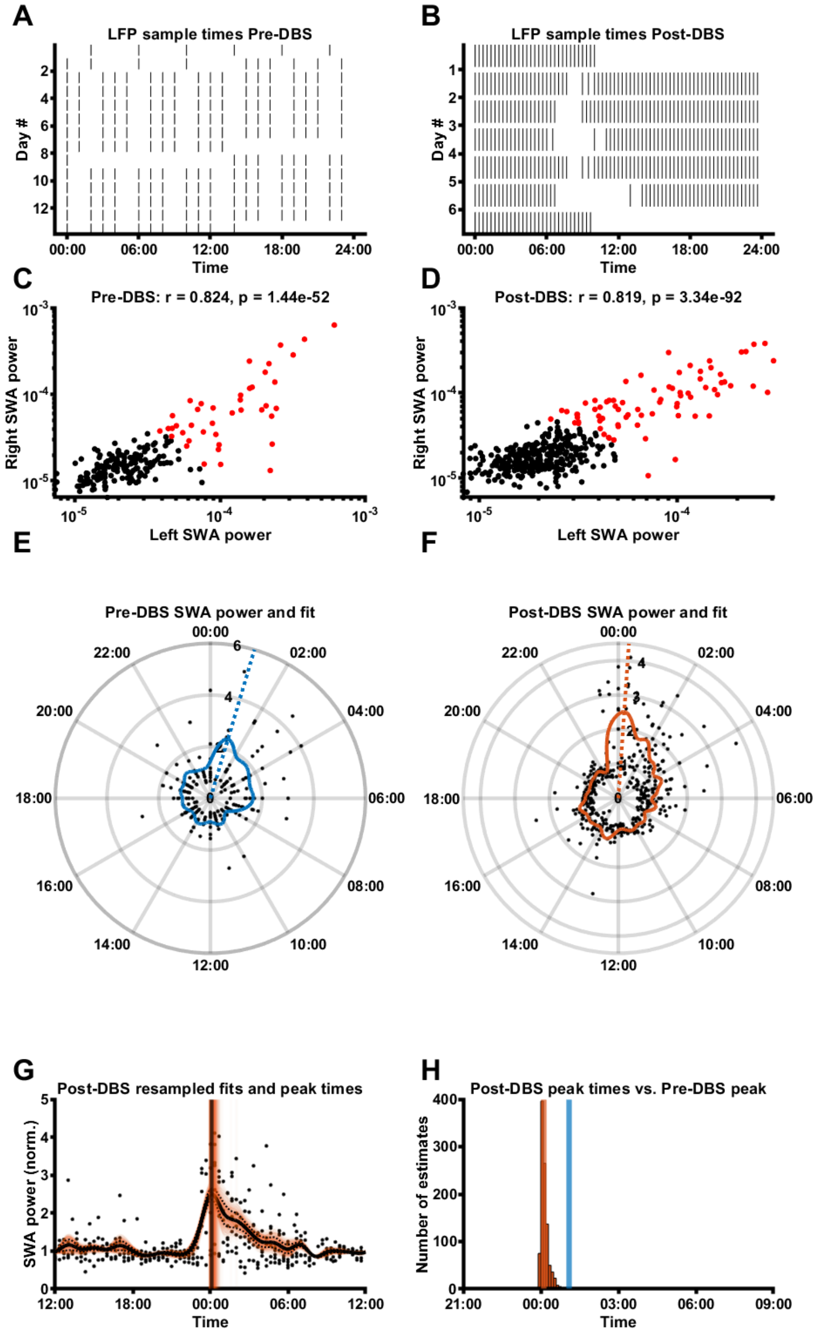

Figure S8: P007 SWA data overview

A, B: SCC LFP sample times in the Pre-DBS (A) and Post-DBS (B) phase. **C, D:** Correlation of left and right SCC SWA power Pre-DBS (C) and Post-DBS (D). **E, F:** SWA power (normalised to median) of all LFPs for this participant (mean across valid hemispheres), plotted around the 24h diurnal cycle. The coloured line represents a smoothing spline fit to the data; the dashed line represents the time of the maximum night-time (18:00-10:00) peak of the SWA fit. **G:** SWA power (normalised to median) of all LFPs for this participant (mean across valid hemispheres), with a mean±SD fit line (solid black line and dashed black lines) superimposed on 1000 fit lines (thin, orange) obtained through random re-sampling of Post-DBS data according to the sample times Pre-DBS (see Methods for details). Also indicated is the median fit peak estimate (black vertical line) superimposed on 1000 fit peak estimate lines (thin orange vertical lines). **H:** Distribution of fit peak time estimates obtained through the 1000 random resamples of the Post-DBS phase, with the median peak time estimate indicated with the orange vertical line and the fit peak time from Phase B indicated with the blue vertical line.

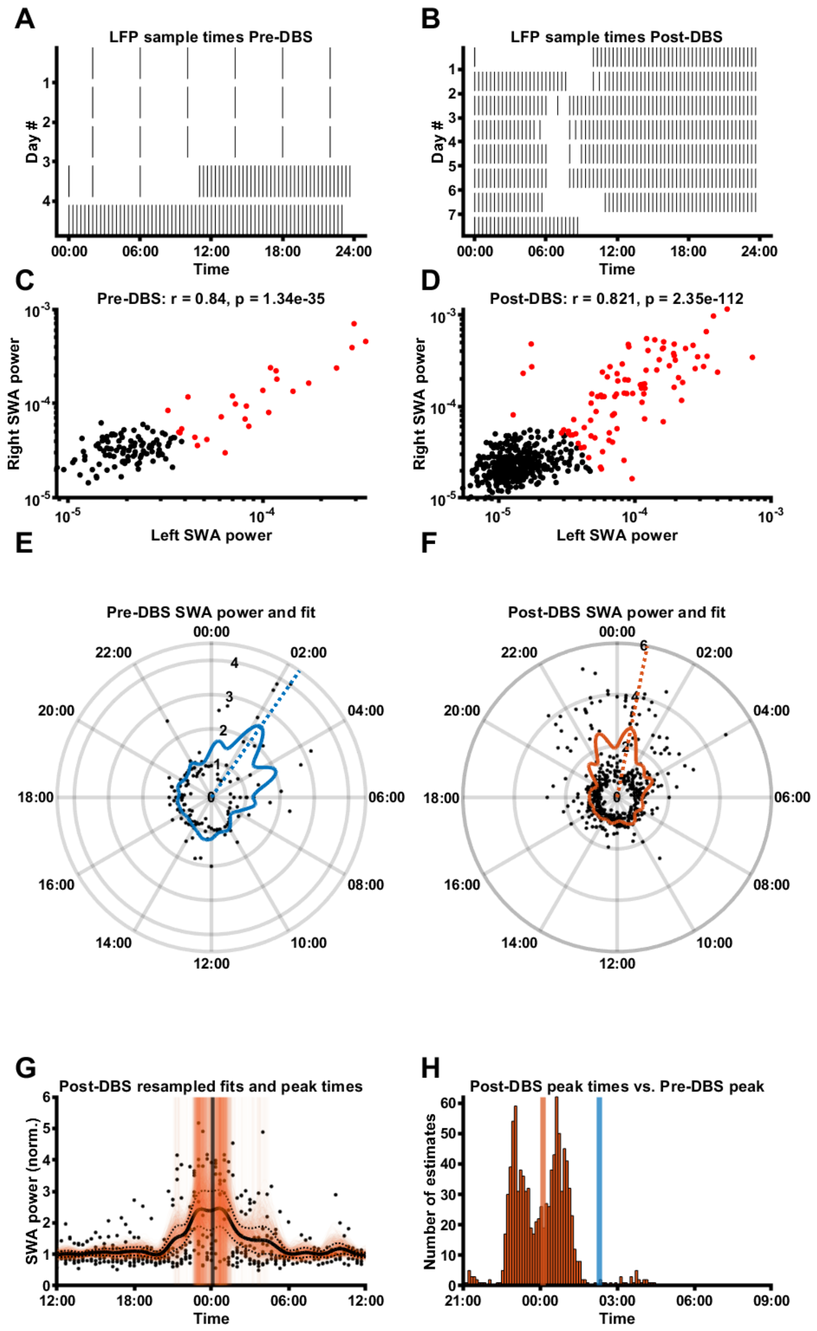

Figure S9: P008 SWA data overview

A, B: SCC LFP sample times in the Pre-DBS (A) and Post-DBS (B) phase. **C, D:** Correlation of left and right SCC SWA power Pre-DBS (C) and Post-DBS (D). **E, F:** SWA power (normalised to median) of all LFPs for this participant (mean across valid hemispheres), plotted around the 24h diurnal cycle. The coloured line represents a smoothing spline fit to the data; the dashed line represents the time of the maximum night-time (18:00-10:00) peak of the SWA fit. **G:** SWA power (normalised to median) of all LFPs for this participant (mean across valid hemispheres), with a mean±SD fit line (solid black line and dashed black lines) superimposed on 1000 fit lines (thin, orange) obtained through random re-sampling of Post-DBS data according to the sample times Pre-DBS (see Methods for details). Also indicated is the median fit peak estimate (black vertical line) superimposed on 1000 fit peak estimate lines (thin orange vertical lines). **H:** Distribution of fit peak time estimates obtained through the 1000 random resamples of the Post-DBS phase, with the median peak time estimate indicated with the orange vertical line and the fit peak time from Phase B indicated with the blue vertical line.

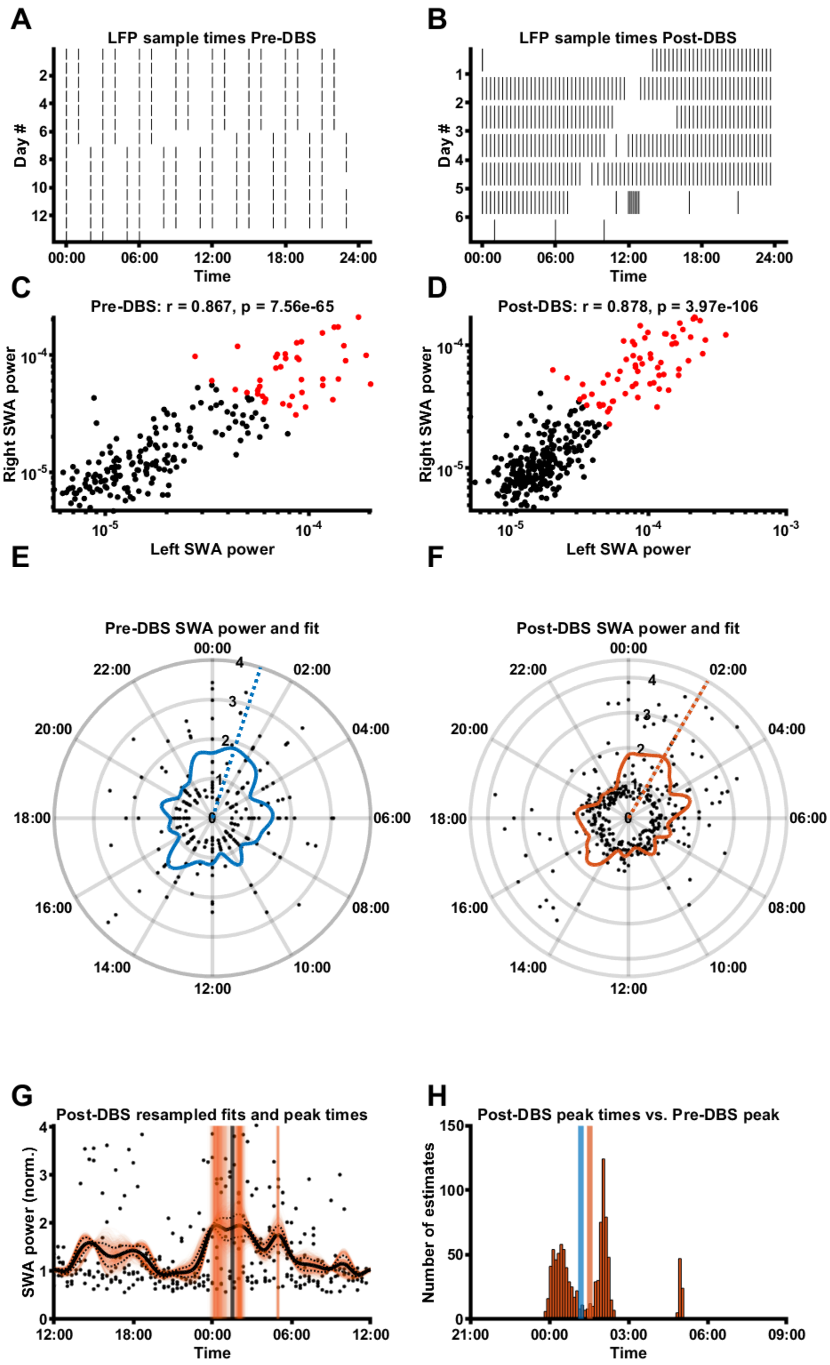

Figure S10: P009 SWA data overview

A, B: SCC LFP sample times in the Pre-DBS (A) and Post-DBS (B) phase. **C, D:** Correlation of left and right SCC SWA power Pre-DBS (C) and Post-DBS (D). **E, F:** SWA power (normalised to median) of all LFPs for this participant (mean across valid hemispheres), plotted around the 24h diurnal cycle. The coloured line represents a smoothing spline fit to the data; the dashed line represents the time of the maximum night-time (18:00-10:00) peak of the SWA fit. **G:** SWA power (normalised to median) of all LFPs for this participant (mean across valid hemispheres), with a mean±SD fit line (solid black line and dashed black lines) superimposed on 1000 fit lines (thin, orange) obtained through random re-sampling of Post-DBS data according to the sample times Pre-DBS (see Methods for details). Also indicated is the median fit peak estimate (black vertical line) superimposed on 1000 fit peak estimate lines (thin orange vertical lines). **H:** Distribution of fit peak time estimates obtained through the 1000 random resamples of the Post-DBS phase, with the median peak time estimate indicated with the orange vertical line and the fit peak time from Phase B indicated with the blue vertical line.

Supplementary tables

Supplementary Table 1: Patient medication schedule (kept constant between study phases)

Patient ID	Medication / supplement	Regime
**P001**	Bupropion extended release 300 mg	qDay
	Bupropion 75 mg	qDay
	Bupropion extended release 150 mg	q24hr
	Cephalexin 500 mg	QID
	Cholecalciferol 2,000 IU	qDay
	Clonazepam 0.5 mg	qHS, PRN
	Ferrous gluconate 325 mg	qDay
	l-methylfolate15 mg	qDay
**P002**	Citalopram	
	Desvenlafaxine 50 mg extended release	qDay
	Docusate 100 mg	BID, PRN
	Lamotrigine 200 mg	
	Ondansetron 4 mg	q8hr, PRN
**P003**	Clonazepam 2 mg	qHS
	Dextromethorphan 30 mg	qDay
	Diazepam 5 mg	q8hr, PRN
	Diltiazem 180 mg	qDay
	Diphenhydramine 25 mg	qHS
	Docusate 100 mg	q12hr
	Escitalopram 40 mg	qDay
	Melatonin 5 mg	qHS
	Omega-3 polyunsaturated fatty acids 1,000 mg	TID
	Ondansetron 4 mg	q8hr, PRN
	Acetaminophen/aspirin/caffeine (Excedrin) 2 tab(s)	q6hr
**P004**	Lunesta 3mg	qHS,PRN
	Gabapentin 300 mg	TID
	l-methylfolate	qDay
	Fetzima 120 mg,	qDay
	Ativan 1 mg,	BID, PRN
	Quetiapine 200 mg	qPM
	Tramadol 50 mg	q4hr
**P005**	Clonazepam 1.5 mg	qHS
	Levothyroxine 175 mcg	qDay
	Nicotine 1 patch(es), Topical	q24hr
	Nortriptyline 150 mg	qHS
	Quetiapine 300 mg	qHS
	Sertraline 50 mg	qDay
	Sertraline 100 mg	qDay
**P006**	Amitriptyline 350 mg	
	Lorazepam 1 mg	TID, PRN
**P007**	Adderall 30mg	
	Clonazepam 1 mg	
	Pristiq	qDay
**P008**	Bupropion 450 mg	q24hr
	Lunesta 1 mg,	qHS, PRN
	Lorazepam 0.5 mg	BID, PRN
	Trokendi XR 200 mg	qDay
**P009**	Klonopin 0.5 mg	BID
	Cymbalta 120 mg	qDay
	Lithium 300 mg	

Supplementary Table 2: Pearson's correlation coefficients of Pre-DBS Hamilton scores and sleep subscales vs. SWA fit peak time and height. Bonferroni-corrected alpha level = 0.01.

	SWA peak time		SWA peak height	
	r	*p* (*α*=0.01)	*r*	*p* (*α*=0.01)
**Hamilton score**	-0.567	0.143	0.418	0.302
**Initial insomnia**	-0.439	0.276	-0.027	0.950
**Midnight insomnia**	-0.310	0.455	0.278	0.506
**Morning insomnia**	-0.501	0.206	-0.074	0.862
**Hypersomnia**	-0.132	0.755	0.341	0.408

Supplementary Table 3: Pearson's correlation coefficients of the Pre- vs. Post-DBS difference in Hamilton scores and sleep subscales vs. the Pre- vs. Post-DBS difference in SWA fit peak time and height. Bonferroni-corrected alpha level = 0.01.

	Δ SWA peak time		Δ SWA peak height	
	r	*p* (*α*=0.01)	*r*	*p* (*α*=0.01)
**Δ Hamilton score**	0.004	0.993	0.130	0.759
**Δ Initial insomnia**	-0.204	0.628	-0.061	0.885
**Δ Midnight insomnia**	0.022	0.958	-0.359	0.382
**Δ Morning insomnia**	-0.636	0.090	-0.071	0.867
**Δ Hypersomnia**	-0.158	0.709	0.712	0.048

Supplementary Table 4: Pearson's correlation coefficients of the Pre- vs. Post-DBS difference in Hamilton scores and sleep subscales vs. the Pre- vs. Post-DBS difference in spindle density and amplitude. Bonferroni-corrected alpha level = 0.01.

	Δ Spindle density		Δ Spindle amplitude	
	r	*p* (*α*=0.01)	*r*	*p* (*α*=0.01)
**Δ Hamilton score**	0.231	0.583	0.236	0.574
**Δ Initial insomnia**	-0.579	0.133	0.188	0.656
**Δ Midnight insomnia**	0.481	0.228	0.117	0.783
**Δ Morning insomnia**	-0.152	0.719	-0.458	0.254
**Δ Hypersomnia**	0.498	0.209	0.214	0.611
